# Circuit Mechanisms of L-DOPA-Induced Dyskinesia (LID)

**DOI:** 10.3389/fnins.2021.614412

**Published:** 2021-03-10

**Authors:** Kai Yang, Xinyue Zhao, Changcai Wang, Cheng Zeng, Yan Luo, Taolei Sun

**Affiliations:** ^1^School of Chemistry, Chemical Engineering and Life Science, Wuhan University of Technology, Wuhan, China; ^2^Department of Physiology, School of Basic Medical Science, Ningxia Medical University, Yinchuan, China; ^3^State Key Laboratory of Advanced Technology for Materials Synthesis and Processing, Wuhan University of Technology, Wuhan, China

**Keywords:** neuronal oscillation, firing pattern, firing rate, Parkinson’s disease, L-DOPA induced dyskinesia

## Abstract

L-DOPA is the criterion standard of treatment for Parkinson disease. Although it alleviates some of the Parkinsonian symptoms, long-term treatment induces L-DOPA–induced dyskinesia (LID). Several theoretical models including the firing rate model, the firing pattern model, and the ensemble model are proposed to explain the mechanisms of LID. The “firing rate model” proposes that decreasing the mean firing rates of the output nuclei of basal ganglia (BG) including the globus pallidus internal segment and substantia nigra reticulata, along the BG pathways, induces dyskinesia. The “firing pattern model” claimed that abnormal firing pattern of a single unit activity and local field potentials may disturb the information processing in the BG, resulting in dyskinesia. The “ensemble model” described that dyskinesia symptoms might represent a distributed impairment involving many brain regions, but the number of activated neurons in the striatum correlated most strongly with dyskinesia severity. Extensive evidence for circuit mechanisms in driving LID symptoms has also been presented. LID is a multisystem disease that affects wide areas of the brain. Brain regions including the striatum, the pallidal–subthalamic network, the motor cortex, the thalamus, and the cerebellum are all involved in the pathophysiology of LID. In addition, although both amantadine and deep brain stimulation help reduce LID, these approaches have complications that limit their wide use, and a novel antidyskinetic drug is strongly needed; these require us to understand the circuit mechanism of LID more deeply.

## The Introduction of L-DOPA–Induced Dyskinesia

Parkinson disease (PD) is a neurodegenerative disorder that occurs often in the elderly. Most of its motor symptoms are caused by the progressive death of dopaminergic (DAergic) neurons in substantia nigra pars compacta (SNc) and deficiency of DA in the striatum (Poewe et al., 2017). Currently, DA replacement therapy using L-3,4-dihydroxyphenylalanine (L-DOPA) is the standard treatment for PD patients. However, its long-term administration usually induces L-DOPA–induced dyskinesia (LID) in the majority of PD patients (Duvoisin, 1967; Turcano et al., 2018; Kim et al., 2020). The antiparkinsonian efficacy of L-DOPA is closely coupled with dyskinesia. Once LID is established, it is difficult to alleviate these dyskinetic symptoms without the compromise of its antiparkinsonian efficacy. The main factors associated with the development of dyskinesia include the disease duration and the age at onset of PD. Many studies have shown that longer disease duration with greater disease severity of PD is associated with a higher risk of LID (Rajput et al., 2002; Sharma et al., 2010). In addition, younger age at disease onset is more likely to develop LID (Kostic et al., 1991; Sharma et al., 2010).

However, these risk factors seem insufficient to explain the incidence of dyskinesia in PD patients; there is some evidence indicating that genetic factors may also contribute to the occurrence of dyskinesia. They include opioid receptor (Strong et al., 2006), brain-derived neurotrophic factor (Foltynie et al., 2009), solute carrier family 6 member 3 (Kaplan et al., 2014), and catechol-*O*-methyltransferase Val58Met (de Lau et al., 2012). But these genetic components are not generally considered primary pathophysiology mechanism for LID. The importance of these genetic factors in the overall risk of developing LID needs further study. Recently, using genomics, transcriptomics, and proteomics methods, it has suggested that expression of one or more specific molecular triggers may induce long-term adaptations of striatal circuits, which result in LID; one of them is nuclear receptor related 1 (Nurr1) protein (Sellnow et al., 2020; Steece-Collier et al., 2020).

Despite significant advances, the pathogenesis of LID remains incompletely understood. It is well accepted that LID is caused by the combination of nigral denervation and chronic pulsatile DAergic receptor stimulation, which establishes inappropriate signaling between the motor cortex and the striatum, contributing to the generation of dyskinesia.

A pathophysiological interpretation of LID implicates both presynaptic and postsynaptic changes in DA transmission. In PD, as the disease progresses, most of SNc DAergic neurons die, and its ability to control extracellular DA in the brain is impaired. In this situation, the majority of the conversion from L-DOPA to DA occurs in serotonergic neurons. But different from DAergic neurons, serotonergic neurons cannot modulate the release of DA, which results in the fluctuation of DA levels at the synaptic clefts (for reviews, see Huot et al., 2013; Bastide et al., 2015). In addition, the DAergic denervation in the dorsolateral striatum leads to a supersensitivity of DA receptors in the striatum, which strongly stimulates cAMP signaling pathway (Park et al., 2014; Sancesario et al., 2014) and DA- and cAMP-regulated neuronal phosphoprotein (DARPP-32) pathway (Guan et al., 2007; Santini et al., 2007). Other signaling cascades are also activated, including ERK kinase (Fasano et al., 2010; Santini et al., 2010) and the mammalian target of rapamycin (Santini et al., 2009; Calabrese et al., 2020). These pathways regulate gene transcription and protein synthesis, which contribute to LID generation (for review, see Spigolon and Fisone, 2018).

In addition, emerging evidence has shown that non-DAergic systems including glutamatergic system is also involved in LID pathophysiology (for review, see Cenci, 2014). Microdialysis studies in rodent models of LID have revealed that extracellular levels of glutamate in the striatum are increased (Dupre et al., 2011). Furthermore, amantadine, a weak non-competitive *N*-methyl-D-aspartate receptor (NMDAR) antagonist, is the first drug approved for dyskinesia by the Food and Drug Administration. Interestingly, activity-dependent synaptic plasticity at corticostriatal synapses is altered in LID, demonstrating its inability to form both LTD (long-term depression) and depotentiation in dyskinesia (Picconi et al., 2003, 2008).

Another non-DAergic system, the cholinergic system, also contributes to LID development. In the striatum, the principal source of acetylcholine is the cholinergic interneurons (ChIs). ChIs have widespread axonal arborizations to modulate striatal neurotransmission (Calabresi et al., 2000). This modulation could influence striatal DA, γ-aminobutyric acid (GABA), and other neurotransmitter release via nicotinic and muscarinic acetylcholine receptors (nAChRs and mAChRs) (for reviews, see Conti et al., 2018; Bordia and Perez, 2019). It has shown that acute L-DOPA administration increases ERK phosphorylation in spiny projection neurons (SPNs), whereas repeated application leads to the activation of ERK in ChIs, which leads to increased basal firing and potentiated responses to DA in ChIs (Ding et al., 2011). In addition, both pharmacological inhibition of striatal cholinergic tone and ablation of striatal ChIs decrease LID without affecting the beneficial efficacy of L-DOPA (Ding et al., 2011; Won et al., 2014).

Furthermore, accumulating evidence indicates that morphologic changes in dendritic spines may also underlie dyskinesia (Fieblinger and Cenci, 2015; Nishijima et al., 2018). In Parkinsonian rodents, the treatment of L-DOPA could induce a remarkable structural plasticity of striatal spines. Spines in indirect pathway SPN (iSPN) are lost after a DA-denervating lesion, but they can regrow in response to the application of L-DOPA; this spine regrowth exhibits aberrant morphology, indicating that the affected iSPNs are abnormal rewired (Zhang et al., 2013). In contrast, direct pathway SPN (dSPN) spine density is reduced (Fieblinger et al., 2014; Nishijima et al., 2014). But how the specific spine changes of iSPNs and dSPNs are related to the pathophysiology of LID remains unknown.

In this review, we will first evaluate three models to explain the pathophysiology of LID, including “firing rate,” “firing pattern,” and “ensemble” model. We will present an overview of studies supporting or negating these models. Then we will discuss brain regions, which are involved in LID, indicating that the generation and modulation of LID require the coordinated actions of the striatum, the pallidal–subthalamic network, the motor cortex, the thalamus, and the cerebellum. Finally, we provide three critical questions for the future study.

## Theoretic Models of BG in LID

**FIGURE 1:**
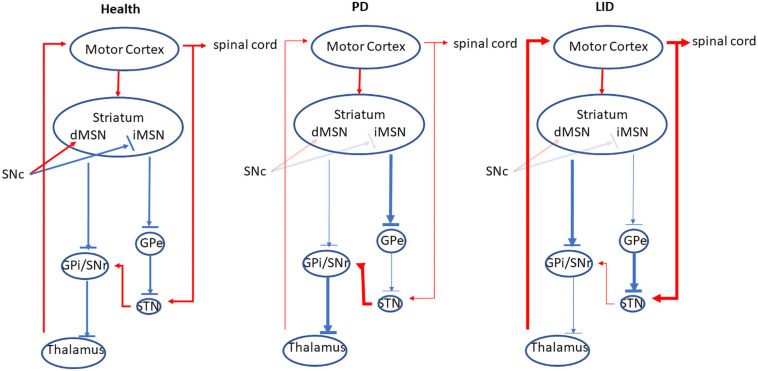
The direct and indirect pathway of the BG. In the health, the activation of the direct pathway reduces the output of the BG, stimulating the thalamus and cortex, which promotes the movement. While the stimulation of the indirect pathway increases the BG output, suppressing the cortex and inhibiting the movement. In PD, the deficiency of DA in the striatum causes the overactivation of indirect pathway, as well as the hypoactivation of direct pathway, resulting in the suppression of the thalamus and the cortex, which inhibits the movement. In contrast, in LID, the activity of direct output pathway is increased while the indirect output pathway is inhibited, which disinhibits the thalamus and the cortex, resulting in the increase of the movement. Red lines indicate excitatory transmission, whereas blue lines mean inhibitory transmission, and line width represents the strength of neuronal transmission. This figure adapted from McGregor and Nelson (2019).

There are two major populations of cells in the striatum, dSPNs and iSPNs, based on their different projection targets. DA D_1_ receptors are highly expressed in dSPNs; these neurons project directly to the outputs of the basal ganglia (BG) including substantia nigra reticulata (SNr) and the globus pallidus internal segment (GPi). In contrast, DA D_2_ receptors are located in iSPNs. They reach SNr and/or GPi indirectly via globus pallidus external segment (GPe) and the subthalamic nucleus (STN) (for review, see Bastide et al., 2015). Generally, the activation of the direct pathway reduces the output of the BG, stimulating the thalamus and cortex, which promotes the movement. While the stimulation of the indirect pathway increases the BG output, suppressing the cortex and the movement (for review, see Bastide et al., 2015; Figure 1). In PD, the deficiency of DA in the striatum causes the overactivation of indirect pathway, as well as the hypoactivation of direct pathway, resulting in the suppression of the thalamus and the cortex, which inhibits the movement (Figure 1; McGregor and Nelson, 2019). While in LID the DA concentration in the striatum is increased, which enhances the output of direct pathway by the stimulation of D_1_ receptor, the indirect pathway is inhibited via the activation of D_2_ receptor. The overall outcome of these actions reduces the BG output and induces dyskinesia (Figure 1).

There are 3 major hypotheses that explain the pathophysiology of LID. First, the “firing rate model” proposes that decreasing the mean firing rates of the output nuclei of BG including GPi and SNr, along the BG pathways, induces dyskinesia. Second, “firing pattern model” claimed that abnormal firing pattern of single unit activity and local field potentials (LFPs) may disturb the information processing in the BG, resulting in dyskinesia. Third, “ensemble model” described that dyskinetic symptoms are induced by alterations in patterns of activity of several specific cell types brain-wide.

### Firing Rate Model (Table 1)

The firing rate model proposes that brain information is encoded in the firing rate of individual neurons. LID symptoms might be caused by the reduction of firing rate of GPi neurons, which disinhibit the thalamic–cortical motor pathway (DeLong, 1990; Vitek and Giroux, 2000). Evidences favoring rate-based model of LID came from studies in humans, non-human primates, and rodents. In Parkinsonian monkeys, the application of L-DOPA reduces the firing rate in the GPi neurons (Papa et al., 1999). Further, in PD patients with LID, during the expression of dyskinesias, both GPi and STN firing rate shift from increased activity in the Parkinsonian state to hypoactivity (Lozano et al., 2000). Studies in rodents with LID also support rate-based model. In 6-OHDA–lesioned rats, the firing rate of STN neurons was decreased by L-DOPA and non-selective DA receptor agonist apomorphine (Kreiss et al., 1997).

**TABLE 1:** Changes in firing rates in health, PD, and LID conditions.

	Parkinsonian	LID	Citations
**Striatum**			
dMSNs	Decreased	Increased	Parker et al., 2018; Ryan et al., 2018; Sagot et al., 2018
iMSNs	Increased	Decreased	Parker et al., 2018; Ryan et al., 2018; Sagot et al., 2018
ChIs	NT	Enhanced	Ding et al., 2011
**STN**	Increased	No effect	Aristieta et al., 2012
**SNr**	Increased	Decreased	Aristieta et al., 2016; Jin et al., 2016
**M1 Cortex**	Decreased	Increased	Ueno et al., 2014
**LC**	Decreased	Normal	Miguelez et al., 2011
**LHb**	NT	Increased	Bastide et al., 2014
**DRN**	Increased	Increased	Prinz et al., 2013

*Simple depiction of firing rate changes in health, PD, and LID conditions across many brain regions including the BG. “NT” means “not tested.”*

Recently, firing rate changes of dSPN and iSPN in LID are also studied. Ryan et al. (2018) showed that, in Parkinsonian mice, DA depletion significantly reduces dSPN firing rates, whereas the firing rate of iSPNs is not increased significantly, which cause an imbalance between dSPN and iSPN activity. After the administration of L-DOPA, the firing rate of dSPNs in Parkinsonian mice is much higher than that in healthy controls, whereas the firing rate of iSPNs is reduced below the normal range (Ryan et al., 2018). In addition, using Pitx3 null mutant (Pitx3^–/–^) mice, which is an animal model for PD with severe DA denervation, the firing rate of dSPNs is increased in response to L-DOPA or D_1_ receptor agonist, SKF81297 (Sagot et al., 2018). A recent study using imaging somatic Ca^2+^ dynamics also reveals that during dyskinesia, dSPNs are hyperactive, whereas iSPNs are hypoactive (Parker et al., 2018).

In addition, chronic L-DOPA administration enhanced baseline and DA-induced firing rates compared with chronic saline treatment in striatal ChIs of Pitx3^–/–^ mice (Ding et al., 2011). The follow-up study by the same group showed that both hyperpolarization–activation cyclic nucleotide-gated and small conductance calcium-activated potassium (SK) channels mediate the change of ChI firing rates in response to chronic L-DOPA treatment (Choi et al., 2020).

Although many studies have favored the rate-based model of LID, it still cannot explain several aspects of LID. For example, by monitoring thousands of SPNs in behaving mice during LID, four different types of SPN dynamics have been revealed, including decreased spontaneous iSPN activity, increased spontaneous dSPN activity, loss of spatial coordination of dSPN activity, and reduced movement-coupled dSPN activity (Parker et al., 2018). Both decreased iSPN activity and increased dSPN activity support the rate-based model, but two other kinds of neural dynamics cannot be explained by this model (Parker et al., 2018).

### Firing Pattern Model

#### Burst Firing

Although many investigators attribute altered firing rates to the generation of LID at the neuronal level in the BG and other brain areas, recent studies have challenged this idea. It now seems clear that changes in burst firing might underlie these dyskinetic symptoms (Table 2). Burst firing means a neuron repeatedly fires discrete groups of spikes; between bursts is a period of quiescence. At least two main functional roles of bursts have been proposed. First, bursting can enhance the reliability of information transmission (Lisman, 1997). Second, bursts can carry additional information and expand the coding space.

**TABLE 2:** Comparisons of firing patterns in health, PD, and LID conditions.

	Parkinsonian	LID	Citations
**Burst firing**			
SNr	NT	Increased	Meissner et al., 2006
	Increased	Reduced	Aristieta et al., 2016
GPi	NT	Increased	Jin et al., 2016
	NT	Increased	Levy et al., 2001a
STN	NT	Increased	Levy et al., 2001a
LC	Decreased	Increased	Miguelez et al., 2011
LHb	NT	Increased	Bastide et al., 2016
**Neuronal oscillation**			
β oscillation			
STN	Increased	Decreased	Delaville et al., 2015
	Increased	Decreased	Alonso-Frech et al., 2006
Cortex	Increased	Decreased	Delaville et al., 2015
	NT	Decreased	Dupre et al., 2016
γ oscillation			
Cortex	Reduced	Increased	Delaville et al., 2015
	NT	Increased	Dupre et al., 2016
	NT	Increased	Swann et al., 2016
	NT	Increased	Halje et al., 2012
STN	Reduced	Increased	Delaville et al., 2015

*The summary of firing patterns (burst firing and oscillation) changes in health, PD, and LID conditions. “NT” means “not tested.”*

At the level of BG output, several groups have found altered bursting activity in GPi neurons in human dyskinesia patients and rodents with LID. Acute application of L-DOPA increases the number of bursting cells in the GPi and SNr of Parkinsonian rats (Meissner et al., 2006; Jin et al., 2016). In contrast, in another study, although baseline bursting firing pattern is increased in Parkinsonian rats, acute application of L-DOPA reduces the number of bursting neurons in the SNr of dyskinetic rats (Aristieta et al., 2016). Increased bursting has also been observed in the STN and GPi of dyskinetic patients (Levy et al., 2001a).

In addition to the BG, neurons in other brain areas also demonstrated changes in burst activity in dyskinetic rodents. In locus coeruleus (LC), the percentage of neurons with bursting activity is significantly reduced in Parkinsonian rats, but acute L-DOPA administration increases it (Miguelez et al., 2011). Furthermore, in lateral habenula (LHb) of ON-L-DOPA dyskinetic 6-OHDA–lesioned rats, the proportion of bursting neurons are significantly increased compared to the control (Bastide et al., 2016).

As with firing rate, it is difficult to link bursting specifically with dyskinesia. Measuring dyskinesia by the modulation of bursting is a good approach. Although one study has shown that bursting increase by constant positive current injection in the STN ameliorates LID in Parkinsonian rats (Tai et al., 2020), more work still needs to be done to confirm this link.

#### Neuronal Oscillations

Convergent evidence from both human patients and animal models suggest that dyskinetic symptoms might also be caused by abnormal changes in neuronal oscillation within the cortico–BG–thalamic loop and other brain regions (Table 2) (Richter et al., 2013).

Mechanisms that mediate the generation of oscillation in brain regions are prime candidates for the pathophysiology of LID.

Neuronal oscillations are rhythmic or repetitive patterns of neural activity in the central nervous system. Brain can generate oscillatory activity driven either by individual neurons or by interactions between neurons. For individual neurons, oscillations can appear as rhythmic patterns of action potentials. At the level of cell groups, synchronized activity of large numbers of neurons produces macroscopic oscillations, which can be measured using LFP. It generally arises from feedback interactions between the neurons. Usually, it is classified into several bands based on frequency range, including δ (∼1–4 Hz), θ (∼4–8 Hz), α (∼8–13 Hz), β (∼13–30 Hz), and γ (∼30–100 Hz) bands (Wang, 2010). The oscillatory theory is an important theoretic basis for deep brain stimulation (DBS) therapy on LID (Stefani et al., 2019). There is a wide diversity of cellular and circuit mechanisms underlying the generation of these oscillations, and neural oscillations could mediate memory, sleep, motor coordination, and other physiological functions.

#### β Oscillation

Most of studies have demonstrated that enhanced β oscillation in cortico–BG–thalamic loop might cause motor impairment including bradykinesia in PD (Brazhnik et al., 2012, 2014, 2016; Delaville et al., 2015). Correlative evidence showed that β frequency stimulation at the STN may contribute to the slowing of movements in PD patients (Chen et al., 2007). It is postulated that in Parkinsonian state the BG demonstrates abnormally β oscillatory activities; this β band activity impairs information processing in cortico–BG loops and causes both motor and cognitive deficits in PD.

**FIGURE 2:**
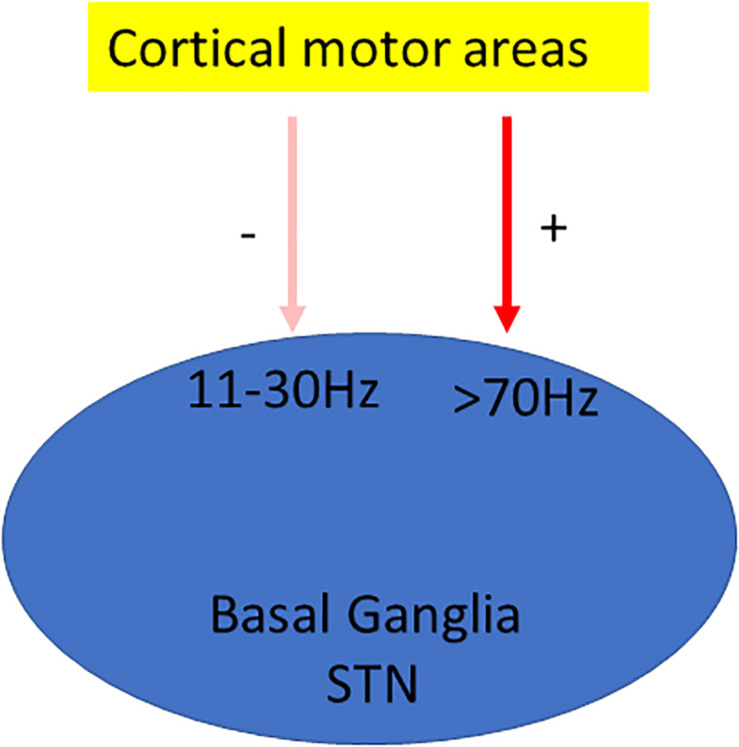
Oscillation model of LID. Oscillation model proposes that dyskinesia is characterized by significant changes in oscillations; usually β oscillation is reduced (Delaville et al., 2015), whereas γ oscillation is enhanced (Halje et al., 2012).

In dyskinetic rodents and patients β band activity is reduced (Figure 2). Treatment with L-DOPA, administrated acutely or chronically, reduces β band activity induced by DA depletion (Delaville et al., 2015). Additionally, in Parkinsonian rats, L-DOPA priming decreases cortical β band activity during treadmill walking (Dupre et al., 2016). PD patients in the “On” state also showed decreased β oscillation (Alonso-Frech et al., 2006). In addition, β oscillations can be divided into low (12–19 Hz) and high (19–30 Hz) β band. They have different origins within the cortico–BG–thalamic circuit (Brittain et al., 2014). Low β band shows a greater decrease in power than the high β component in response to L-DOPA and apomorphine (Priori et al., 2004).

Nevertheless, the link between PD and β oscillation is not convincing. In Parkinsonian monkeys injected with 1-methyl-4-Phenyl-1,2,3,6-tetrahydropyridine (MPTP), they show that Parkinsonian symptoms do not depend on β oscillation. While the moderate depletion of DA induces parkinsonism, only a significant DA reduction generates β oscillation (Leblois et al., 2007). Furthermore, the pharmacological inhibition of STN activity by microinjection of lidocaine and muscimol into STN can rescue some PD symptoms without suppressing β oscillation in PD patients (Levy et al., 2001b). Similarly, how the change of β oscillation is related to dyskinesia requires further study.

#### γ Oscillation

γ Oscillation is also associated with dyskinesia. Initial studies suggested that this oscillation is primarily related to the prokinetic effect of L-DOPA rather than to dyskinetic symptoms (Cassidy et al., 2002; Alonso-Frech et al., 2006). It is further supported by the study showing that the power of γ oscillation increases with voluntary movements (Cassidy et al., 2002; Alegre et al., 2005). The high-frequency oscillations (HFOs) in the 60–90-Hz frequency band are also found in the motor cortex in association with movements (Crone et al., 1998). Interestingly, these movements related only to brief episodes of γ power increase rather than sustained oscillatory activity (Cheyne and Ferrari, 2013). Later, the link of this HFO to dyskinesia in motor cortex is established by Halje et al. (2012) (Figure 2). They found that 80-Hz HFOs are present only in the lesioned hemisphere during LID. Further, the local application of D_1_ receptor antagonist in the motor cortex reduces both 80-Hz oscillation and dyskinesia (Halje et al., 2012).

Since the original finding by Halje et al., the role of this narrowband γ oscillation in rodent models of LID has been further confirmed (Dupre et al., 2016; Swann et al., 2016). In PD patients with an implantable bidirectional device for DBS and electrocorticography, this typical motor cortical HFOs is also observed along with dyskinesia, supporting the conclusion that this narrowband HFO is pathological rather than prokinetic (Swann et al., 2016). In addition, this oscillation is not affected by voluntary movements, indicating that it can be a reliable biomarker of dyskinesia (Swann et al., 2016). Furthermore, the appearance of γ oscillation is related to the severity of DA damage as the partially lesioned rats do not show the enhancement of narrow γ band activity, this oscillation is obvious when LID becomes severe (Dupre et al., 2016). Both γ oscillation and dyskinesia can be induced by activating either D_1_ or D_2_ dopamine receptors similar to that induced by L-DOPA following L-DOPA priming in 6-OHDA–lesioned rats (Dupre et al., 2016).

Although an association between fast cortical oscillations and LID is strongly suggested, it is still controversial. In the ventral striatum of healthy rodents, the application of apomorphine and amphetamine, which are DA agonists, also increases high γ activity (∼80 Hz) (Berke, 2009).

It is well accepted that slow oscillations are necessary for network synchronization over long distances, whereas faster rhythms including γ rhythms serve to synchronize neuronal activity in short ranges (Jensen and Colgin, 2007). The amplitude of γ oscillation could be modulated by the phase of a much slower oscillation, which is called phase-amplitude coupling. It has been demonstrated in many brain areas and plays very important roles in a variety of cognitive processes including learning and memory (Canolty and Knight, 2010). Recently, phase-amplitude coupling has also been discovered in both the cortex and the striatum of mice with dyskinesia. In the case of dyskinesia, the coupling of the amplitude of oscillation at ∼80 Hz to the phase of low frequencies is significantly reduced (Belic et al., 2016).

### Ensemble Model

A neural ensemble is a group of neurons involved in a special neural computation. Depending on functional requirements, the same neuron could participate in different cell ensembles related to different computations (Josselyn et al., 2015). There is anatomical evidence to support this notion that each neuron receives inputs from many other neurons while sending its outputs to large populations of cells. It is proposed that dyskinesia symptoms might represent a distributed impairment involving many brain regions.

It is well accepted that LID is caused by aberrant activity in the striatum evoked by L-DOPA. However, other brain regions have also been involved, including the primary motor cortex (M1) and the cerebellum. It is more likely that dyskinetic symptoms are induced by the alterations in large populations of neurons across the brain, although the number of activated neurons in the striatum correlated most strongly with dyskinesia severity.

Using the targeted recombination in active population technique, neurons in several brain regions, including the M1 and the striatum have been identified to be associated with LID (Girasole et al., 2018). Optical activation of LID-associated striatal neurons induces dyskinesia without the acute administration of L-DOPA. Further, the inhibition of these striatal LID-associated neurons reduces the generation of dyskinesia, whereas inhibiting dSPNs only cannot (Girasole et al., 2018). In another study, a subpopulation of dSPNs, which has abnormally high firing rates induced by L-DOPA in the striatum, is correlated specifically with dyskinesia (Ryan et al., 2018). Interestingly, it has been shown that parvalbumin-positive interneurons and iSPNs in the striatum might also contribute to LID (Girasole et al., 2018), which is supported by another study (Alberico et al., 2017).

## Possible Loci of Circuit Modulation in LID

It is well accepted that the striatum is the major target for L-DOPA to induce LID. In Parkinsonian rat, local administration of L-DOPA in the striatum induces dyskinesia (Buck et al., 2010). Further immediate-early genes such as c-Fos, FosB, and ΔFosB are consistently upregulated in the striatum of animals with LID (Cenci, 2002; Beck et al., 2019). Detailed information about the involvement of the striatum in LID can be found in other articles (Cenci et al., 2018; Zhai et al., 2019). However, other brain regions have also been implicated in LID, including the pallidal–subthalamic network, the motor cortex, the thalamus, and the cerebellum (Figure 2) (Cenci et al., 2018). In addition, a systematic mapping of the brain regions reveals that several other regions including the LHb and the bed nucleus of the stria terminals (BNST) are also involved in LID (Bastide et al., 2014).

### The Pallidal–Subthalamic Network

The pallidal–subthalamic network consists of the STN and the two segments of the globus pallidus (GPe and GPi). Each component of this network has its own set of afferent and efferent connections. The STN is a key structure in the cortico–BG–thalamo-cortical circuit. It receives glutamatergic inputs from the cortex (Heikenfeld et al., 2020), the thalamus (Kita et al., 2016), and the superior colliculus (SC) (Coizet et al., 2009). In addition, it has GABAergic input from the GPe (Canteras et al., 1990) and DAergic input from the SN (Cragg et al., 2004). Furthermore, the STN provides glutamatergic efferent to the outputs of the BG including GPi, SNr, and the striatum (Hamani et al., 2017). It is involved in both indirect pathway and hyperdirect pathway in the cortico–BG–thalamo-cortical circuit (Bonnevie and Zaghloul, 2019). In hyperdirect pathway, STN receives direct signals from the cerebral cortex without the involvement of GPe (Polyakova et al., 2020).

STN lesion or STN high-frequency stimulation (HFS) alleviates dyskinesia by reducing the dose of the L-DOPA required (Toda et al., 2004; Apetauerova et al., 2006; Aristieta et al., 2012). The optogenetic inhibition of the STN significantly reduced LID in Parkinsonian rats (Yoon et al., 2016). However, STN-HFS may also produce dyskinesia in some PD patients (Limousin et al., 1996) and Parkinsonian rodents (Beurrier et al., 1997; Boulet et al., 2006). Furthermore, after LID in Parkinsonian rats is induced, the depletion of DA in the STN significantly attenuates LID, but the removal of DA in the STN alone could not induce dyskinesia in Parkinsonian rats (Marin et al., 2013).

The GPi is an output of the BG, which receive afferent stimulation from striatal dSPN directly. The efferent of the GPi includes the thalamus (Jaeger and Kita, 2011). Another GP, the GPe, receives two major inputs from STN and striatal iSPNs (Jaeger and Kita, 2011). Part of the direct targets from the GPe includes the STN, the striatum, and the thalamus (Mastro et al., 2014). Different cell groups in the GPe connect to its different targets. Prototypical neurons in the GPe send strong projections to the STN, whereas arkypallidal neurons project to both SPNs and fast-spiking interneurons in the striatum (Gittis et al., 2014). Several studies have shown that the GPi is involved in LID. Chronic DBS of GPi improves dyskinesia in Parkinsonian rats (Rodrigues et al., 2007; Alam et al., 2014). In contrast to STN stimulation, the stimulation of the GPi could improve dyskinesia without changing the requirement for L-DOPA (Apetauerova et al., 2006). Up now, no study investigates the involvement of GPe in LID.

### Substantia Nigra Pars Reticulata

The SN is divided into two parts: SNc and SNr. The pars compacta serves mainly as a projection to the BG circuit, supplying the striatum with DA. The pars reticulata conveys signals from the BG to numerous other brain structures. Similar to GPi, the neurons in pars reticulata are mainly GABAergic.

SNr receives several afferents from other brain regions, including GABAergic inputs from the striatum and glutamatergic projections from the STN. SNr also sends significant connections to the thalamus and SC (Beckstead et al., 1979). In addition, the neurons in SNr form collaterals with pars compacta, modulating the activity of DAergic neurons in the pars compacta (Mailly et al., 2003).

Many studies have shown that SNr is involved in LID. During dyskinesia, the activities of SNr neurons in patients and animal models of PD are significantly inhibited (Lozano et al., 2000; Meissner et al., 2006; Aristieta et al., 2016). In addition, optical stimulation of dSPN GABAergic terminals at the SNr can produce a full dyskinetic state similar to that induced by L-DOPA (Keifman et al., 2019). Interestingly, the stimulation of M4 mAChRs in SNr inhibits LID (Brugnoli et al., 2020).

### Motor Cortex

The motor cortex comprises three different areas, including the M1, the premotor cortex, and the supplementary motor area (SMA) (Bastide et al., 2015). Accumulated evidence indicated that the motor cortex is involved in LID (Huang et al., 2011; Halje et al., 2012; Belic et al., 2016; Dupre et al., 2016). Most of studies have been done in PD patients and rodents. It has shown that LID is associated with increased activity of neurons in M1 (Lindenbach et al., 2016). Using single-photon emission computed tomography technique, both SMA and M1 are found to be overactivated in PD patients with LID by analyzing regional cerebral blood flow (Rascol et al., 1998). This conclusion has also been confirmed by magnetic resonance imaging data (Cerasa et al., 2015). In addition, in Parkinsonian rats, the application of L-DOPA increases M1 blood flow while decreasing M1 glucose metabolism (Ohlin et al., 2012). But other studies reported different results; in PD patients, L-DOPA reduces M1 blood oxygen levels and glucose metabolism (Haslinger et al., 2001). These inconsistent studies require future clarification.

In the rodent brain, pyramidal cells account for 80% of cortical neurons. There are two different types of pyramidal neurons in the motor cortex: intratelencephalic (IT) and pyramidal tract (PT) neurons based on their projection targets (Reiner et al., 2010). IT neurons preferentially target both ipsilateral striatum and contralateral striatum as well as the cortex. Therefore, IT neurons include both corticostriatal and corticocortical projections, whereas PT neurons preferentially connect brainstem, spinal cord, and ipsilateral striatum (McColgan et al., 2020). In addition, it has been suggested that IT neurons express D_1_ receptors and preferentially target dSPNs, whereas PT neurons express D_2_ receptors and preferentially connect iSPN in the striatum (Shepherd, 2013), but this hypothesis is challenged by other studies; it has shown that IT and PT neurons project to both iSPN and dSPN in the striatum (Kress et al., 2013). Further work is required to study this inconsistency. In LID rat model, both IT-type and PT-type neurons in the M1 demonstrate enlarged dendrite spines (Ueno et al., 2014, 2017). IT-type neurons also show increased neuronal activity (Ueno et al., 2014); whether the activity of PT-type neurons is also increased remains unstudied yet (Ueno et al., 2017).

Using transcranial magnetic stimulation (TMS), a lack of depotentiation-like cortical plasticity in PD patients with LID is also revealed (Huang et al., 2011). In the motor cortex of LID rat model, 80-Hz high-frequency LFP oscillations appear (Halje et al., 2012). The association between high-frequency LFP oscillations and LID has also been confirmed by several other studies (Delaville et al., 2015; Belic et al., 2016; Dupre et al., 2016). In addition, when D_1_ receptors in the motor cortex is pharmacologically inhibited, both 80-Hz oscillations and abnormal involuntary movement (AIM) are attenuated (Halje et al., 2012). Thus, not surprisingly, directly targeting the M1 with TMS and transcranial direct current stimulation (tDCS) has been used to treat LID symptoms. M1-tDCS improves LID symptoms in PD patients, which might be mediated by downregulating M1 excitability (Ferrucci et al., 2016).

### Thalamus

In addition to act as a relay afferent to the neocortex, the thalamus is also related to the movement control. It includes several important nuclei such as the centromedian/parafascicular complex (CM/Pf) and the motor thalamus. CM/Pf can communicate bidirectionally with the BG; CM/Pf provides major glutamatergic inputs to the BG (Van der Werf et al., 2002). In turn, it receives innervations from the BG output (Sidibe et al., 2002). Further, CM/Pf has wide afferent connections from sensorimotor cortex and responses to many sensory and arousing stimuli (Yamanaka et al., 2018). There is accumulating evidence demonstrating the involvement of CM/Pf in LID (Jouve et al., 2010; Alam et al., 2014). In 6-OHDA–lesioned Parkinsonian rat model, Pf-HFS partially alleviates LID. In addition, Pf-HFS can also provide antiparkinsonian benefits, although its efficacy is reduced by L-DOPA (Jouve et al., 2010). At the cellular level, Pf-HFS partially prevents the increase of preproenkephalin-A mRNA levels in the striatum induced by DA denervation, preproenkephalin-A is a marker for iSPN function (Jouve et al., 2010). Further, Pf-HFS reverses the lesion-induced changes of cytochrome oxidase subunit I in the STN, GPe, and SNr (Jouve et al., 2010). Chronic DBS of CM/Pf improves the dyskinetic symptoms in both Parkinsonian rats and patients (Stefani et al., 2009; Alam et al., 2014), although one study showed that CM/Pf lesion in MPTP-lesioned dyskinetic monkeys provides no benefit on LID symptoms (Lanciego et al., 2008). Further studies are still required to understand the specific contribution of CM/Pf neurons to the pathophysiology of LID.

Another nucleus in the thalamus, the motor thalamus, is also linked to LID, which is demonstrated by several studies (Ohye and Shibazaki, 2001; Guridi et al., 2008). It includes the ventral anterior, ventral lateral, and ventral medial nuclei. They receive direct afferents from GPi/SNr, the cerebral cortex, and the deep cerebellar nuclei. The lesions of the motor thalamus improve dyskinetic symptoms in PD patients with LID (Ohye and Shibazaki, 2001; Guridi et al., 2008).

### Cerebellum

**FIGURE 3:**
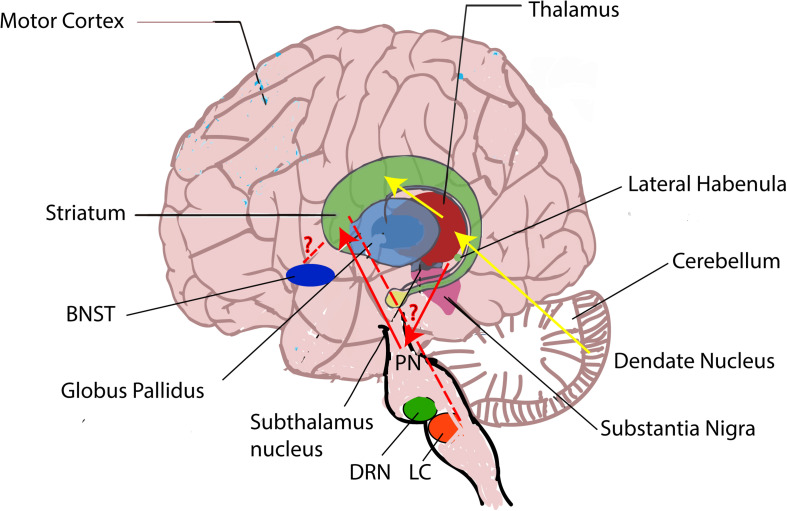
Brain regions involved in the pathophysiology of LID. Besides the striatum, other brain regions including the pallidal–subthalamic network, the motor cortex, the thalamus, the cerebellum, the dorsal raphe nucleus (DRN), the LC, the LHb, and the BNST are also involved in LID. During LID, LHb neurons are suggested to induce the aberrant DA release in the striatum from serotonergic terminals via DRN and generate LID symptoms (Carta et al., 2007; Bernard and Veh, 2012), but the mechanisms how the LC and the BNST affect LID remain unknown (red “?” represents unknown). In addition, the BG and the cerebellum are thought to communicate at the subcortical level. The subthalamic nucleus (STN) sends projections to the cerebellar cortex through pontine nuclei (PN) (blue line) (Bostan et al., 2010), whereas the dentate nucleus in the cerebellum sends connection to the striatum via the thalamus (yellow line) (Hoshi et al., 2005).

Although both the BG and the cerebellum are involved in the movement control, they have different functions. The function of the cerebellum is to control fine-tuning movement, whereas BG’s function is to select wanted movement. These two systems are thought to be independent, and they connect only at the level of the cerebral cortex. But studies have shown that the BG and the cerebellum could also communicate at the subcortical level (Hoshi et al., 2005; Bostan et al., 2010). The STN sends projections to the cerebellar cortex through pontine nuclei (Bostan et al., 2010), whereas the dentate nucleus in the cerebellum sends connection to the striatum via the thalamus (Hoshi et al., 2005; Figure 3).

Most studies demonstrating cerebellar involvement in LID are done in human beings using tDCS and/or TMS. For example, cerebellar tDCS decreases LID in PD patients (Ferrucci et al., 2016). It is proposed that the cerebellum is overactivated in LID. This impairs its ability for efficient information processing. Cerebellar tDCS reduces LID by decreasing the overstimulation of cerebellum (Ferrucci et al., 2016). In addition, repetitive TMS is used on the lateral cerebellum in PD patients with LID. It has shown that a single session of cerebellar continuous theta burst stimulation (TBS) transiently reduces LID, whereas multiple sessions of cerebellar TBS alleviate LID for a longer time (Koch et al., 2009). Cerebellar TBS might do so by decreasing short intracortical inhibition and increasing long intracortical inhibition, which reorganizes cortical circuits linked to LTD induction (Koch et al., 2009).

Furthermore, a single session of inhibitory cerebellar TMS could rescue the deficits of sensorimotor M1 plasticity in PD patients with LID, whereas repeated cerebellar TMS had an antidyskinetic effect along with a resurgence of sensorimotor plasticity in M1, suggesting this effect of cerebellar stimulation could restore M1 plasticity (Kishore et al., 2014). It is hypothesized that the abnormal output from the BG might affect cerebellar sensory processing function, which induces both the maladaptive plastic responses in M1 and the appearance of LID (Kishore et al., 2014).

### Other Brain Regions

#### Lateral Habenula

LHb receives innervations from both the limbic system and the BG including GPi (Hikosaka et al., 2008; Hong and Hikosaka, 2008). It projects to the monoaminergic brain regions such as the ventral tegmental area (VTA), the SNc, and the dorsal and medial raphe (DRN and MRN) (Bernard and Veh, 2012). Recent study claims that LHb might encode negative reward, which is involved in reward processing (Baker et al., 2016). So far, only one article demonstrates that LHb contributes to the generation of LID. It demonstrates that L-DOPA increases LHb activity. In addition, LID symptoms are relieved when LHb neurons are inactivated (Bastide et al., 2016). It is well known that LHb projects to serotonin neurons in the DRN. In advanced PD, instead of DAergic terminals, serotonergic terminals control the release of DA. Therefore, during LID, LHb neurons might induce the aberrant DA release from serotonergic terminals and generate LID symptoms (Carta et al., 2007; Navailles et al., 2011; Figure 3).

### Bed Nucleus of the Stria Terminal

BNST is related to motivated behaviors and has two-way communications with the central amygdale, the lateral hypothalamus, the VTA, and the periaqueductal gray (Vranjkovic et al., 2017). It has shown that transcription factors, ΔFosB, ARC, Zif268, and FRA2, are overexpressed in the BNST of dyskinetic rats (Cenci, 2002; Lindgren et al., 2011; Bastide et al., 2014). When neurons in BNST are pharmacogenetically inactivated, dyskinesia is significantly reduced (Bastide et al., 2017). It is hypothesized that the BNST could modulate LID symptoms via SNc and that it might also affect LID severity, either directly or indirectly, through the modulation of anxiety-related and sensorimotor processes.

### Dorsal Raphe Nucleus

DRN contains the main population of serotonergic neurons in the brain; they provide extensive innervation to the BG including SN and caudate putamen (Di Matteo et al., 2008). In rats, both the number of serotonergic neurons in the DRN and serotonin content in the striatum and prefrontal cortex are significantly reduced after the administration of L-DOPA; these changes might be mediated by oxidative-stress mechanism (Stansley and Yamamoto, 2014). Consistently, the decrease in serotonin content after the application of L-DOPA in a rat model of PD is also observed; but different from previous study, this reduction occurs throughout the whole rat brain (Navailles et al., 2011). These differences might be caused by different experimental models and methods they used. But other authors found the opposite. This is thought to be a sprouting of serotoninergic neurons induced by L-DOPA in Parkinsonian rat, which potentiates the DA release in the DA-depleted striatum (Rylander et al., 2010). Interestingly, there are no anatomical changes for the serotonergic neurons in the DRN of PD patients with LID, suggesting a functional but not structural change in the serotonergic system in dyskinesia (Cheshire et al., 2015).

In addition, both DA degeneration and subsequent L-DOPA treatment affect the intrinsic excitability of serotonergic neurons in the DRN of Parkinsonian mouse model (Prinz et al., 2013). This change might mediate some dyskinetic symptoms. But another study drew a different conclusion; they find that the effect of L-DOPA is not related to changes of the activity of rat DRN serotonergic neurons (Miguelez et al., 2016).

Further, several studies have shown that lesions of the rat DRN rescue LID symptoms (Carta et al., 2007; Eskow et al., 2009). The expression of the dopamine D_2_ autoreceptor in serotonergic neurons of rat DRN also blocks LID (Sellnow et al., 2019). Interestingly, the serotonergic and DAergic systems interact reciprocally. The impairment of serotonergic neuron reduces DA release (Navailles et al., 2011), whereas the administration of L-DOPA decreases the level of serotonin (Navailles et al., 2011).

### Locus Coeruleus

It has been shown that noradrenergic (NAergic) neurons in the LC are also degenerated in PD (Del Tredici et al., 2002). In fact, more neurons die in the LC than in the SNc (Zarow et al., 2003). Additionally, it is proposed that the degeneration of LC NAergic neurons happens early before that of SNc DAergic neurons (Braak and Del Tredici, 2008). To date, there is growing evidence that additional loss of NA neurons of the LC, the main source of NA in the brain, can affect LID symptoms (Fulceri et al., 2007; Perez et al., 2007, 2009; Miguelez et al., 2011), but results are not consistent, NA loss can increase, reduce, or have no effect on LID symptoms. Studies have reported that the denervation of LC NAergic terminals increases LID symptoms induced by L-DOPA in Parkinsonian rats (Fulceri et al., 2007; Perez et al., 2007). In addition, chemical destruction of LC increases dyskinesia induced by L-DOPA (Miguelez et al., 2011). In contrast, NA loss does not significantly modulate dyskinetic symptoms in Parkinsonian rats; instead, it reduces therapeutic effects of L-DOPA (Ostock et al., 2014). In another study, NA loss reduces dyskinesia (Barnum et al., 2012).

In addition, the expression of α2A adrenoceptor RNA in the LC is increased in Parkinsonian rats, and long-term treatment of L-DOPA reverses this increase (Alachkar et al., 2012). Further, both selective agonists and antagonists of α2-NAergic receptors modulate dyskinetic symptoms (Ostock et al., 2015). Systemic and local LC infusions of clonidine, a α2-NAergic receptor agonist, reduces LID and locomotor activity without modulating L-DOPA’s antiparkinsonian benefits. Conversely, the application of atipamezole, a specific α2-NAergic receptor antagonist, prolongs LID (Ostock et al., 2015). It is proposed that atipamezole might modulate motor function indirectly by stimulating the release of NA and/or inhibiting the activity of α2-NAergic receptor at postsynaptic site. NAergic system might help to remove DA derived from L-DOPA via the NA transporter (Arai et al., 2008). In addition, when both the NAergic and DAergic systems are denervated, L-DOPA-derived DA remains for a long time in the cleft, which leads to a prolongation of dyskinesia (Hida and Arai, 1998).

## Conclusion

In this review, based on accumulated evidence, we proposed that LID-related changes of network activity including burst firing and oscillation of neurons might induce dyskinesia. While firing rates clearly change across the BG–thalamus–cortex loop and other brain areas in both LID patients and animal models, extensive evidence suggests that dyskinesia is characterized by significant changes in burst firing and oscillations.

Despite the accumulation of extensive evidence for circuit mechanisms in driving LID symptoms, several major questions remain.

(1) Do changes in burst firing or oscillation cause dyskinesia? Or are they just epiphenomenon? Most evidence supporting their roles in dyskinesia is correlative, but no matter whether they are epiphenomenon or causal, they can provide important biological marker for the dyskinesia diagnosis. This could facilitate drug screening studies in PD rodents with dyskinesia, as well as identifying the better stimulation regimes for PD patients with LID.

(2) In addition, so far it is still difficult to link specific changes in neuronal activity of dyskinesia-related brain regions to dyskinesia. The pathophysiological mechanisms underlying the development of dyskinesias still need further study.
(3) Do current therapies including anti-LID compounds and DBS affect the circuit mechanisms of LID? Several studies have addressed this possibility. For example, the application of amantadine in Parkinsonian mice with dyskinesia reduces high γ oscillation in both M1 and dorsolateral striatum (Zheng et al., 2020). In another study, amantadine increases STN firing rate (Allers et al., 2005). In addition, both the depletion of 5-HT using pCPA (a serotonin synthesis inhibitor) and the blockage of serotonin signaling using its antagonist significantly modify STN neuron firing rate, but whether this modulation mediates the therapy of serotonin ligands on dyskinesia remains unstudied (Aristieta et al., 2014).

## Author Contributions

KY, YL, and XZ reviewed the literature and drafted and wrote the manuscript. CW and CZ revised the manuscript. TS proposed the topic of this manuscript and revised the manuscript. All authors read and approved the final version.

## Conflict of Interest

The authors declare that the research was conducted in the absence of any commercial or financial relationships that could be construed as a potential conflict of interest.
